# Two new temporary ectoparasitic isopods (Cymothoida: Cymothooidea) from Korean waters with a note on geographical distributions of *Rocinela* Leach, 1818 and *Gnathia* Leach, 1814

**DOI:** 10.7717/peerj.14593

**Published:** 2023-01-03

**Authors:** Sung Hoon Kim, Jong Guk Kim, Seong Myeong Yoon

**Affiliations:** 1 Division of Ocean Sciences, Korea Polar Research Institute, Incheon, South Korea; 2 Division of Zoology, Honam National Institute of Biological Resources, Mokpo, South Korea; 3 Educational Research Group for Age-associated Disorder Control Technology, Graduate School, Chosun University, Gwangju, South Korea; 4 Department of Biology, College of Natural Sciences, Chosun University, Gwangju, South Korea

**Keywords:** Ectoparasite, Gnathia, Isopods, Morphology, Rocinela, Taxonomy, South Korea

## Abstract

Two new species of temporary ectoparasitic isopods, *Rocinela excavata* **sp. nov.** and *Gnathia obtusispina* **sp. nov.**, are reported from the southern Islands of the Korean Peninsula. *Rocinela excavata* **sp. nov.** is distinguishable from its related species by the following characteristics: (1) laterally stepped rostrum; (2) separated eyes; (3) propodal blade having eight robust setae; and (4) merus having four or five blunt robust setae in pereopods 1–3. *Gnathia obtusispina* **sp. nov.** differs from its congeners by the combination of the following characteristics: (1) body covered with numerous tubercles and setae, (2) cephalon having tooth-like paraocular ornamentations; and (3) frontal border having two inferior frontolateral processes. These two new species are the 13^th^ *Rocinela* species and 19^th^ *Gnathia* species in the temperate Northern Pacific region, respectively. Discovery of these new species represents high species diversity of the genera *Rocinela* Leach, 1818 and *Gnathia* Leach, 1814 worldwide as well as in the Northern Pacific region. In addition, faunal diversity analysis on the members of both genera revealed that *Rocinela* species show high-latitude diversity whereas *Gnathia* species have low-latitude diversity.

## Introduction

Within isopod taxa, the superfamily Cymothooidea Leach, 1814 including families Aegidae White, 1850 and Gnathiidae Leach, 1814 is predominantly parasites of fish or other crustaceans (Williams & Boyko, 2012; Smit, Bruce & Hadfield, 2019). Among the Cymothooideans, both Aegidae and Gnathiidae are known to be temporary ectoparasites that can attach to fishes (Bruce, 2009; Svavarsson & Bruce, 2012; Williams & Boyko, 2012; Cardoso et al., 2017; Smit, Bruce & Hadfield, 2019). However, aegids are also regarded as free-living micro-predators because they often detach from their hosts and spend most of their time free-living on the seafloor (Bruce, 2009; Williams & Boyko, 2012; Smit, Bruce & Hadfield, 2019). They morphologically differ from other Cymothooideans in terms of the maxillule having robust setae distally, maxillipedal palp articles 3 and 4 having conspicuous recurved robust setae distally, prehensile pereopods 1–3, and ambulatory pereopods 4–7 (Bruce, 2009). Similarly, adults of gnathiid isopods are also free-living (non-feeding) on cryptic habitats of sponges, dead corals, barnacle nests, and polychaete’s tube (Kopuz et al., 2011) whereas their juveniles show a hematophagous life cycle (Svavarsson & Bruce, 2012; Williams & Boyko, 2012; Smit, Bruce & Hadfield, 2019). Although gnathiids show highly polymorphic forms depending on their developmental stages and their adults exhibit considerable sexual dimorphism, they are distinguishable from other cymothooideans largely based on the adult male’s characteristics of having remarkably enlarged mandibles and only five pairs of pereopods (Svavarsson & Bruce, 2012; Ota, 2014; Boxshall & Hayes, 2019; Smit, Bruce & Hadfield, 2019).

So far, seven *Rocinela* species have been recorded from the Far East where the survey region of the present study is located: *Rocinela belliceps* (Stimpson, 1864) from the Sea of Okhotsk, Russia; *Rocinela maculata* Schioedte & Meinert, 1879 from the East Sea, Russia and South Korea; *Rocinela japonica* Richardson, 1898 from the Hakodate Bay, Japan; *Rocinela affinis* Richardson, 1904 from the Shizuoka, Japan; *Rocinela angustata* Richardson, 1904 from the Manazuru, Japan; *Rocinela niponia* Richardson, 1909 from the Sado Island, Japan and Chujado Island, South Korea; and *Rocinela lukini* Vasina, 1993 from the Sea of Okhotsk, Russia (Schioedte & Meinert, 1879; Richardson, 1898, 1904, 1909; Vasina, 1993; National Institute of Biological Resources, 2012; Kim & Yoon, 2020). Eleven *Gnathia* species have been reported in the Far East: *Gnathia tuberculata* Richardson, 1909 from Nanao, Japan; *Gnathia derzhavini* Gurjanova, 1933 from Askold Island, Russia; *Gnathia rectifrons* Gurjanova, 1933 from the East Sea, Russia; *Gnathia schmidti* Gurjanova, 1933 from the Bay of Vladimir, Russia; *Gnathia bungoensis* Nunomura, 1982 from the Saeki Bay, Japan; *Gnathia nasuta* Nunomura, 1992 from Kumamoto and Okinawa Islands, Japan; *Gnathia sanrikuensis* Nunomura, 1998 from the Otsuchi Bay, Japan; *Gnathia capillata* Nunomura & Honma, 2004 from Sado Island, Japan; *Gnathia mutsuensis* Nunomura, 2004 from Asamushi, Japan; *Gnathia gurjanovae* Golovan, 2006 from Peter the Great Bay, Russia; and *Gnathia koreana* Song & Min, 2018 from Geomundo Island, South Korea (Boyko et al., 2008; Song & Min, 2018; Shodipo et al., 2021).

In this study, we report two temporary ectoparasitic isopods from Korean waters with their detailed descriptions and illustrations. Geographical distributions of these two genera are also discussed.

## Materials and Methods

All materials were collected at the bottom of sublittoral zones using a Smith-McIntyre grab and SCUBA diving. *Rocinela* specimens were sampled from sandy-mud flats by using the Smith-McIntyre grab. *Gnathia* specimens were collected from the bryozoans and seaweeds on bedrock. SCUBA diving was used to survey the bedrock of sublittoral zones. These collected materials were immediately fixed in 95% ethyl alcohol and then transferred to the laboratory. Isopods were sorted from the transferred materials and then observed and dissected under a dissecting microscope (Olympus SZH-ILLD, Japan). Measurements and drawings of specimens were conducted with the aid of a drawing tube on a compound microscope (Olympus, BX50, Shinjuku, Tokyo, Japan) or the dissecting microscope. Pencil drawings were digitally scanned, inked, and arranged using a tablet and Adobe Illustrator CS6 as mentioned in Coleman (2003, 2009). All examined type series and additional material were moved into each small glass vial filled with 95% ethanol and deposited at the National Institute of Biological Resource (NIBR), South Korea.

The electronic version of this article in Portable Document Format (PDF) will represent a published work according to the International Commission on Zoological Nomenclature (ICZN), and hence the new names contained in the electronic version are effectively published under that Code from the electronic edition alone. This published work and the nomenclatural acts it contains have been registered in ZooBank, the online registration system for the ICZN. The ZooBank LSIDs (Life Science Identifiers) can be resolved and the associated information viewed through any standard web browser by appending the LSID to the prefix http://zoobank.org/. The LSID for this publication is: (urn:lsid:zoobank.org:pub:7A53937A-F2EB-49C7-B8DA-F0AA36241310). The online version of this work is archived and available from the following digital repositories: PeerJ, PubMed Central and CLOCKSS.

## Results

**Taxonomy**

Order Isopoda Latreille, 1817

Suborder Cymthoida Wägele, 1989

Superfamily Cymothooidea Leach, 1814

Family Aegidae White, 1850

Genus *Rocinela* Leach, 1818

Type species. *Rocinela danmoniensis* Leach, 1818 by monotypy.

Diagnosis. Body typically flat, slightly vaulted dorsally; rostrum blunt, covering all or part of antennular peduncles; eyes large, sometimes fused each other, occupying over 50% width of the cephalon; pleonite 1 not abruptly narrower than pereonite 7; antennule shorter than antenna, with distinct peduncles; mandibular incisor narrow, not divided and denticulate; maxillipedal palp consisting of three articles; maxillipedal endite present; pereopod 1–3 with robust setae on propodus; pleopodal endopods 3–4 without plumose setae marginally; uropodal protopod mesially produce; uropodal rami lamellar; pleoptelson distally rounded (Brusca & France, 1992; Bruce, 2009).

Remarks. Synonymy and diagnosis have been well recognized by Brusca & France (1992) and Bruce (2009) and we followed them in this study. Among the members of this family, the genus *Rocinela* Leach, 1818 is distinguishable from other genera by having pleonite 1 not abruptly narrowing than pereonite 7 and a three-articled maxillipedal palp (Bruce, 2009). Although *Rocinela* species show a quite uniform appearances to each other, the shape of the frontal margin of the cephalon and the pereopodal armature are most helpful in identifying species (Brusca & France, 1992; Bruce, 2009). *Rocinela signata* Schioedte & Meinert, 1879 is one of the rare isopods that is known to attack humans (Garzón-Ferreira, 1990).

*Rocinela excavata* sp.nov.

urn:lsid:zoobank.org:act:9A4CC86D-6930-4FC6-9FC9-DBF105A2B285

Figures 1–3

**Figure 1:**
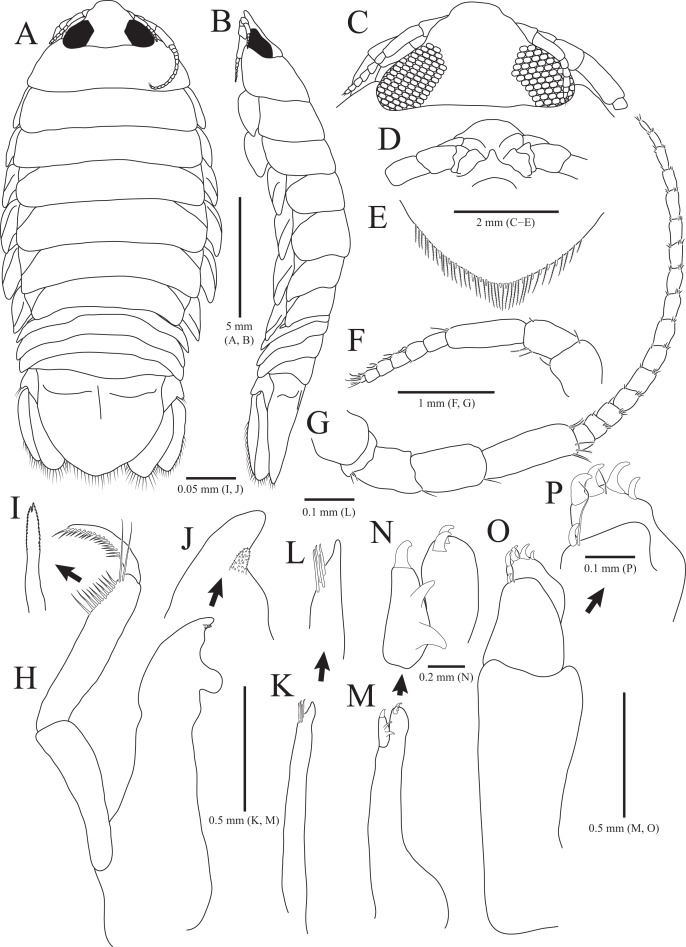
*Rocinela excavata* sp. nov., holotype, male. (A) Habitus, dorsal view; (B) Habitus, lateral view; (C) Cephalon, dorsal view; (D) Distal end of cephalon, ventral view; (E) Distal end of pleotelson; (F) Antennule; (G) Antenna; (H) Mandible; (I) Serrate seta of mandibular palp; (J) mandibular incisor; (K) Maxillule; (L) Distal end of maxillule; (M) Maxilla; (N) Distal end of maxilla; (O) Maxilliped; (P) Distal end of maxilliped. Scale bars: A, B = 5 mm, C–E = 2 mm, F, G = 1 mm; H, K, M, O = 0.5 mm, N = 0.2 mm, L, P = 0.1 mm, I, J = 0.05 mm.

**Figure 2:**
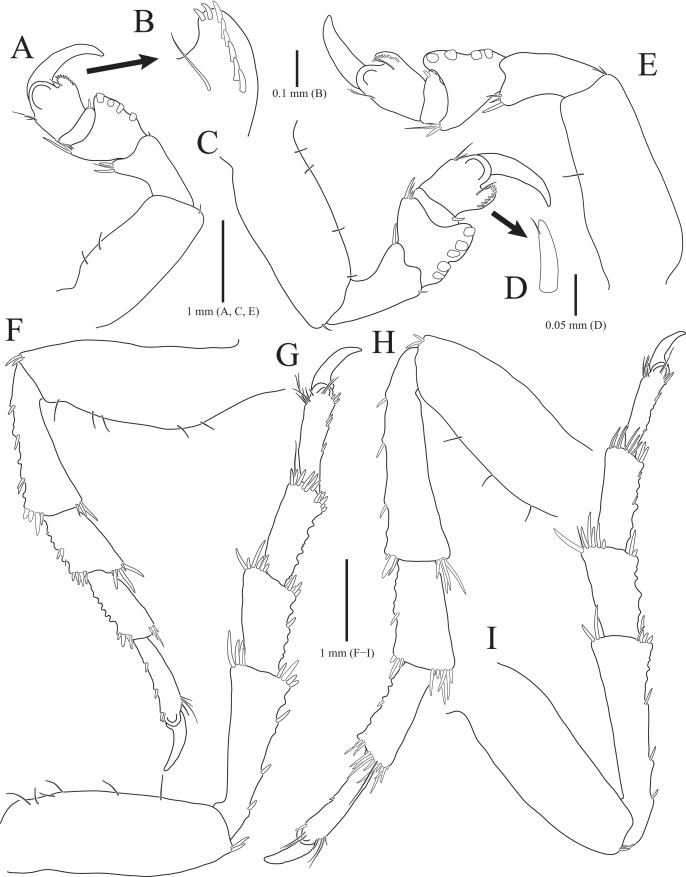
*Rocinela excavata* sp. nov., holotype, male. (A) Pereopod 1; (B) Propodal blade of pereopod 1; (C) Pereopod 2; (D) Robust seta of propodal bladed in pereopod 2; (E) Pereopod 3; (F) Pereopod 4; (G) Pereopod 5; (H) Pereopod 6; (I) Pereopod 7. Scale bars: A, C, E, F–I = 1 mm, B = 0.1 mm; D = 0.05 mm.

**Figure 3:**
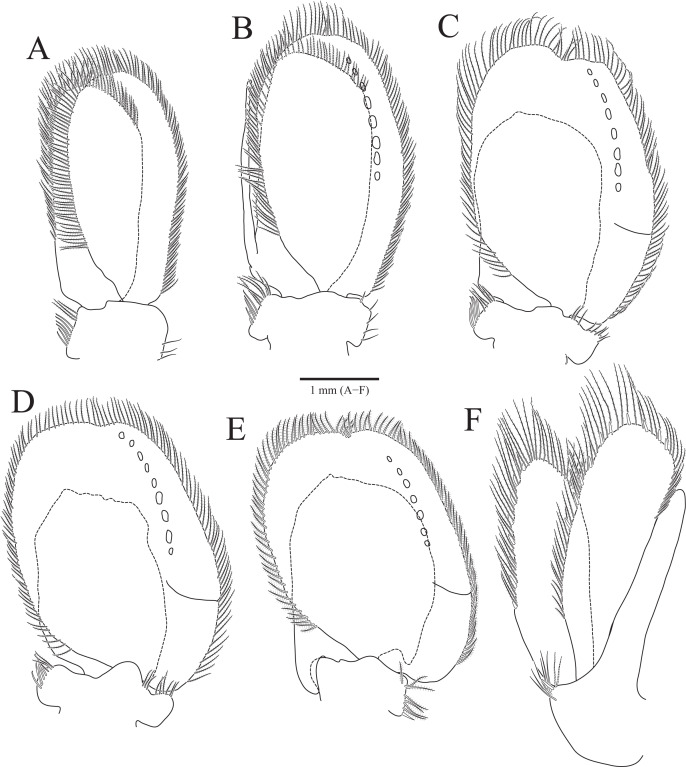
*Rocinela excavata* sp. nov., holotype, male. (A) Pleopod 1; (B) Pleopod 2; (C) Pleopod 3; (D) Pleopod 4; (E) Pleopod 5; (F) Uropod. Scale bar: A–F = 1 mm.

Type material.—Holotype, designated here: South Korea: ♂, 19.3 mm, Chujado Island (33°58′50″N, 126°20′23″E), Chuja-myeon, Jeju-si, Jeju-do, 15 January 2019, 30–40 m, gravelly mud flats, S.H. Kim leg., Smith-McIntyre grab, NIBRIV0000900845. Paratype: 1♂, the same location as holotype, NIBRIV0000895341.

Description of holotype male. Body (Figs. 1A and 1B), 2.1 times longer than width, oval, dorsoventrally depressed; dorsal surface smooth. Cephalon (Figs. 1C and 1D) triangular; posterior margin slightly tri-sinuated, but not distinct; rostrum truncated anteriorly, stepped laterally; eyes large, separate. Pereonite 1 slightly longer than other pereonites; pereonite 3 widest; pereonite 7 narrower than preceding pereonites, tapering posteriorly. Coxal plates visible on dorsal side, acute posteriorly; coxal furrows present in coxal plates 4–7. Pleonite 1 hidden by pereonite 7, slightly visible on both lateral sides; pleonites 2–4 with subacute apex, but pleonite 5 with blunt apex. Pleotelson (Fig. 1E) semicircle or shield-shaped, tapering posteriorly, with numerous plumose setae and robust setae distally; lateral margins concave proximally; dorsal surface with one pair of depressions proximally and one medial carina.

Antennule (Fig. 1F) reaching anterior margin of pereonite 1; peduncular article 1 wider than article 2, with two penicillate setae distally; article 2 subequal to article 1 in length, with three penicillate setae and one simple seta laterally; article 3 elongated oblong, longest, 1.7 times longer than article 2, with one penicillate seta and two short simple setae distally; flagellar article 1 rectangular, 0.3 times as long as peduncular article 3, without setae; articles 2–5 square, with two aesthetascs distally; article 6 min, with two aesthetascs, one penicillate seta, and three simple setae. Antenna (Fig. 1G) exceeding beyond posterior margin of pereonite 1; peduncular article 1 globular; article 2 short, with three simple setae distally; article 3 4.7 times longer than article 2, with one simple seta distally; article 4 oblong, 1.5 times longer than article 3, with one simple seta; article 5 elongated, longest, 1.3 times longer than article 4, with three penicillate setae and three simple setae distally; flagellum consisting of 16 articles; each article with short simple setae distally except for first article without setae.

Frontal lamina (Fig. 1D) short, subacute distally; labrum projecting downwardly. Mandible (Figs. 1H–1J), incisor acute, with one process covered by minute spinous papulae; molar process rounded; palp article 2 longer than others, with 10 serrated setae and two long simple setae along with lateral margin; article 3 with 17 serrate setae (bifurcated distally) laterally. Maxillule (Figs. 1K and 1L) slender, four robust setae distally; apex acute. Maxilla (Figs. 1M and 1N) stout proximally; inner lobe with one curved robust seta distally and two protrusions laterally; outer lobe with two curved robust setae. Maxilliped (Figs. 1O and 1P), first article oblong, 2.6 times longer than width, wider posteriorly; second article 0.3 times as long as first article, with one curved robust seta and long simple seta distally; third article 0.6 times longer than second article, with four curved robust setae and one simple seta distally.

Pereopods 1–3 (Figs. 2A–2E), basis oblong, with 1–4 penicillate setae on superior margin and one simple seta at inferodistal angle; ischium almost 0.5 times as long as basis, expanding superior distal end, with one or two robust setae superodistally; merus trapezoidal, with several robust and simple setae at superodistal angle and four blunt robust setae along with inferior margin, but pereopod 2 with five blunt robust setae; carpus shortest, about 0.3 times as long as merus, with one robust seta on inferodistal end; propodus almost three times longer than carpus, with blade on palm; propodal blade 0.7 times as long as wide, with eight robust setae distally and one long simple seta proximally; robust setae with one simple setule distally; dactylus curved, as long as propodus, without setae. Pereopods 4–7 (Figs. 2F–2I), articles sequentially shortened; basis with 3–6 penicillate setae superiorly and two robust setae inferodistally, longest; ischium to carpus with tubercles and robust setae along with inferior margins, and robust setae at superior distal angles; propodus with several tubercles and robust setae along with inferior margin, and one penicillate seta and several simple setae at superior distal angle; dactylus slightly curved, without setae.

Pleopods (Figs. 3A–3E) sequentially larger posteriorly; pleopods 2–4 with globular patterns along with lateral margins of exopods. Pleopod 1 (Fig. 3A), protopod with six coupling hooks and three plumose setae on medial margin, and three simple setae on lateral margin; rami with plumose setae; exopods slightly longer than endopod. Pleopod 2 (Fig. 3B), protopod rectangular, with five coupling hooks and eight plumose setae medially, one robust seta and four simple setae laterally; endopod smaller than exopod; appendix masculina inserted proximally, expanding distal end of endopod, reaching three-fourths length of endopod. Pleopods 3 and 4 (Figs. 3C and 3D), protopod with coupling hooks and plumose setae on medial margin and plumose setae on lateral margin; endopod much smaller than exopod, without plumose setae; exopod with plumose setae marginally and patch laterally; partial suture present on lateral margin. Pleopod 5 (Fig. 3E) subequal to pleopods 3 and 4, but endopod enlarged beyond protopod and without coupling hooks and plumose setae on medial margin.

Uropod (Figs. 1A, 1B and 3F), reaching distal end of pleotelson; protopod expanding distally on medial margin, one robust seta and eight simple setae on lateral margin; rami elongated oval, with numerous plumose and robust setae; endopod longer than exopod; apexes rounded.

Remarks. The material of *R. excavata* **sp. nov.** can easily be characterized as new to science by the following combinations of characters: (1) the rostrum is truncated anteriorly and stepped laterally; (2) eyes are separated from each other; (3) pereopods 1–3 have eight robust setae on the propodal blade and four or five blunt robust setae on each merus; (4) ischium to carpus in pereopods 4–7 have tubercles along the posterior margins; and (5) one pair of depressions is located at the proximal region of the pleotelson.

Among the known 41 species of the genus *Rocinela*, only three species have separated eyes and more than seven robust setae on the propodal blade in pereopods 1–3: *R*. *niponia* Richardson, 1909, *R*. *garricki* Hurley, 1957, and *R*. *pakari* Bruce, 2009 (Richardson, 1909; Bruce, 2009). Among them, *Rocinela excavata* **sp. nov.** most resembles *R*. *garricki* by sharing characteristics of the rostrum and propodal blade of pereopods 1–3. However, the former can be rapidly distinguished from the latter in terms of the distal end of the rostrum (truncated in the former *vs*. rounded in the latter) and the shape of the robust setae on the merus in pereopods 1–3 (blunt in the former *vs*. subacute in the latter). *Rocinela excavata* **sp. nov.** differs from the *R. niponia* and *R. pakari* in terms of the laterally stepped rostrum (*vs*. not stepped rostrum in the latter two species) and pereopods 4–7 having tubercles along the posterior margins (*vs*. smooth in the latter two species) (Bruce, 2009; Kim & Yoon, 2020).

Among seven species reported from the Far East, *Rocinela excavata* **sp. nov.** is most similar to *R*. *japonica* in the structure of rostrum and setal armature of pereopods 1–3’s merus, while the latter exhibits a distinct difference in the number of setae on the propodal blade in pereopods 1–3 (eight robust setae in the new species *vs*. three or four robust setae in *R*. *japonica*) (Richardson, 1898, 1904, 1909; Kussakin, 1974; Vasina, 1993). *Rocinela excavata* **sp. nov.** can be distinguishable from other six species by having separated eyes (*vs*. fused eyes in *R*. *affinis*), pereopod 1 bearing eight robust setae on the propodal blade (*vs*. less than eight in the latter six other species) (Richardson, 1904, 1909; Kussakin, 1979; Brusca & France, 1992).

Distribution. South Korea (Jeju Strait).

Prey (host). Unknown.

Etymology. The specific name, *excavata*, originates from the combination of Latin prefix *ex-* meaning “out of” and Latin word *cavatus* meaning “hollow out”. It refers to the shape of the rostrum laterally excavated; gender feminine.

Family Gnathiidae Leach, 1814

Genus *Gnathia* Leach, 1814

Type species. *Gnathia termitoides* Leach, 1814 (= *Cancer maxillaris* Montagu, 1804), by monotypy.

Diagnosis. Cephalon with generally straight frontal margin bearing frontal processes, while not deeply concaved; mandibles not elongated, with mandibular incisor and dentate blade; paraocular ornamentation and/or a dorsal sulcus present; pylopod distinct, 2 or 3-articled.

Remarks. Gnathiids show highly polymorphic forms depending on their developmental stages (Ota, 2014; Boxshall & Hayes, 2019). They are distinguishable from other cymothooids largely based on adult male’s characteristics of having remarkably enlarged mandibles and only five pairs of pereopods (Svavarsson & Bruce, 2012; Smit, Bruce & Hadfield, 2019). Among the gnathiids, the genera *Anceus* Risso, 1826, *Praniza* Latreille, 1817, and *Zuphea* Risso, 1826 have been traditionally regarded as junior synonyms of *Gnathia*, because they were based on gnathiid larval stages whose specific identifications cannot be possible (Cohen & Poore, 1994). *Caecognathia* Dollfus, 1901 and *Elahpognathia* Monod, 1926 have been elevated to generic rank by Cohen & Poore (1994). The genus *Gnathia* can be distinguished from others by its male characteristics such as a transverse frontal border on the cephalon having frontal processes, a 2- or 3-articled broad pylopod, and non-elongated mandibles having dentate blades (Cohen & Poore, 1994; Song & Min, 2018; Hadfield et al., 2019).

*Gnathia obtusispina* sp. nov.

urn:lsid:zoobank.org:act:3219C531-9A69-4805-B16D-5B14AF1B9B61.

Figures 4–6

**Figure 4:**
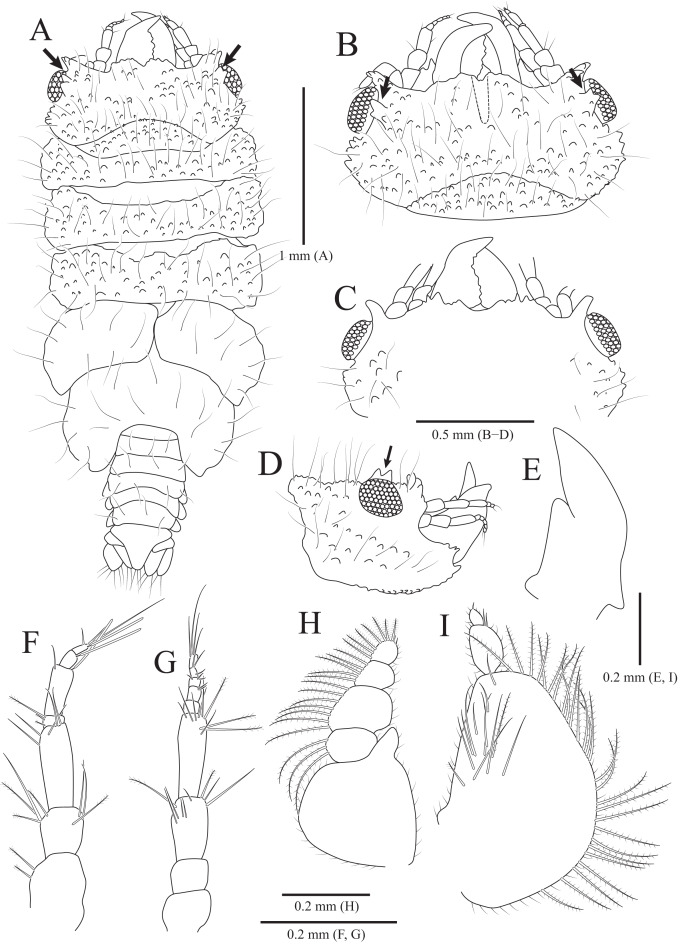
*Gnathia obtusispina* sp. nov., holotype, male. (A) Habitus, dorsal view; (B) Cephalon, dorsal view; (C) Cephalon, ventral view; (D) Cephalon, lateral view; (E) Mandible, lateral view; (F) Antennule; (G) Antenna; (H) Maxilliped; (I) Pylopod. Arrows indicate a tooth-like blunt spine. Scale bars: A = 1 mm, B–D = 0.5 mm, E–I = 0.2 mm.

**Figure 5:**
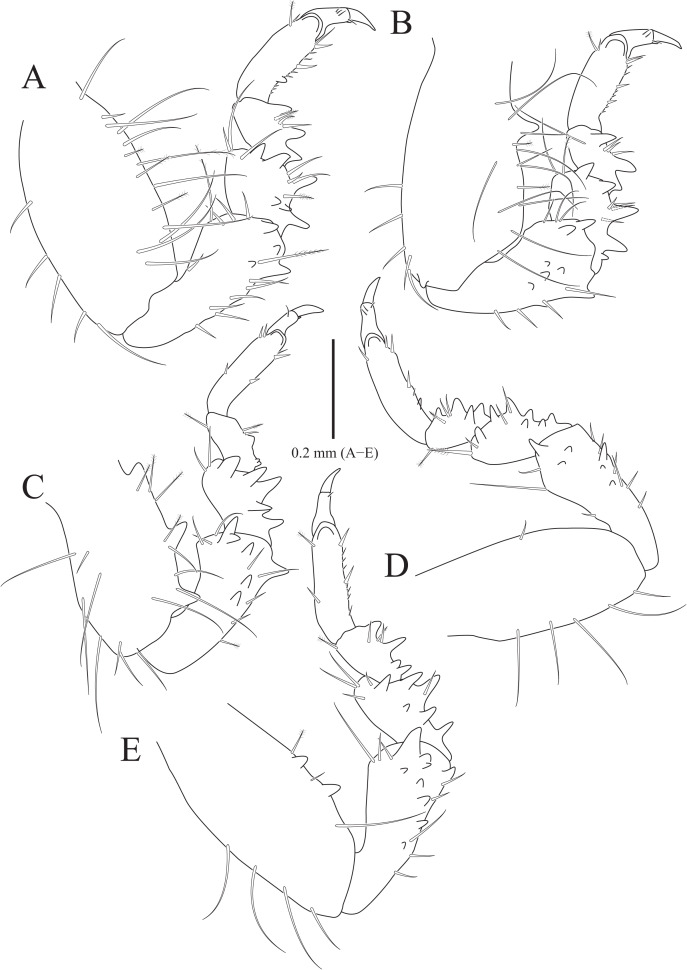
*Gnathia obtusispina* sp. nov., holotype, male. (A) Pereopod 2; (B) Pereopod 3; (C) Pereopod 4, (D) Pereopod 5; (E) Pereopod 6. Scale bar: A–E = 0.2 mm.

**Figure 6:**
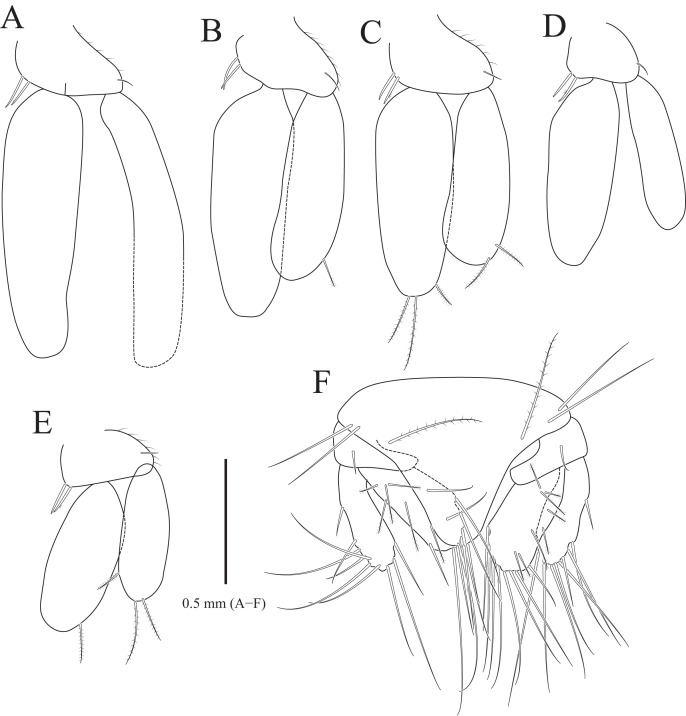
*Gnathia obtusispina* sp. nov., holotype, male. (A) Pleopod 1; (B) Pleopod 2; (C) Pleopod 3; (D) Pleopod 4; (E) Pleopod 5; (F) Pleotelson and uropod. Scale bar: A–F = 0.5 mm.

Type material.—Holotype, designated here: South Korea: ♂, 3.2 mm, Hongdo-ri (34°43′22.8″N, 125°11′59.5″E), Heuksan-myeon, Sinan-gun, Jeollanam-do, 20 June 2018, 10 m depth, rinsing bryozoans and macroalgae on bedrock of sublittoral zones, S. H Kim, leg., SCUBA diving, NIBRIV0000900846. Paratypes: 2♂♂, same location as holotype, NIBRIV0000862802.

Description of holotype male. Body (Fig. 4A) 2.3 times longer than greatest width, with numerous long setae dorsally. Cephalon (Figs. 4B and 4C) oval to oblong, 0.4 times as long as wide, covered with numerous tubercles, with one pair of tooth-like paraocular ornamentations forming ridges (arrows in Figs. 4B and 4C); dorsal sulcus narrow, U-shaped, positioned at median area anteriorly; frontal border medially concave, with one pair of inferior frontolateral processes; frontal concavity shallow and narrow; supraocular lobes prominent, projecting upwards, with dentate apex; eyes located on lateral margins. Pereonites 1–4 covered with tubercles, whereas 5–7 without tubercles; pereonite 1 not fused to cephalon dorsally, immersed in posterior margin of cephalon; pereonites 2–4 subequal in length and width; pereonite 5 widest; pereonite 6 with concave posterior margin. Pleonites, epimera of pleonites 3–5 prominent. Pleotelson (Figs. 4A and 6F) triangular, with convex lateral margins; apex rounded, with three simple setae; proximal dorsal side with two pairs of simple setae and one pair of plumose setae.

Antennule (Fig. 4F), peduncular article 1 ovoid to oblong, with two penicillate setae laterally; article 2 square, 0.7 times longer than article 1, with four penicillate setae and three simple setae distally; article 3 elongate and rectangular, 1.5 times longer than article 2, with five simple setae distally; flagellar article 1 shortest, 0.1 times as long as peduncular article 3, with three penicillate setae laterally; article 2 elongated oblong, 4.9 times longer than article 1, with one simple seta and one aesthetasc distally; article 3 oblong, 0.3 times as long as article 2, with one aesthetasc distally; article 4 subequal to article 3 in length, with three simple setae and one aesthetasc distally. Antenna (Fig. 4G) peduncular article 1 globular; article 2 square, 0.8 times as long as article 1; article 3 oblong, 1.9 times longer than article 2, with two penicillate setae and seven simple setae distally; article 4 elongated rectangular, 1.3 times longer than article 3, with three penicillate setae and six simple setae distally; flagellum composed of five articles; each article square to oblong, similar each other in length, with simple setae distally.

Mandibles (Figs. 4A–4E) triangular, not elongate, half-length of cephalon, elevated distally, with dorsal and internal lobes; dentate blade irregular; basal neck indistinct. Maxilliped (Fig. 4H), endite reaching proximal region of palp article 2; palp articles globular, similar to each other in shape, article 1 with three plumose setae laterally; article 2 largest, with seven plumose setae laterally; article 3 with five plumose setae laterally; article 4 with seven plumose setae laterally and two short simple setae distally. Pylopod (Fig. 4I), article 1 longest, nearly occupying 70% of total length of pylopod, with numerous plumose setae on lateral margin, and 1 penicillate seta, one plumose seta, and 12 simple setae on medioventral side; article 2 ovoid, 0.2 times as long as article 1, with two short simple setae distally; article 1 elliptical, 0.3 times as long as article 2, with one simple seta on distal end.

Pereopod 2 (Fig. 5A) with tubercles on ischium to propodus inferiorly; basis with three penicillate setae superiorly, numerous simple setae superiorly and inferiorly; ischium 0.8 times as long as basis, with one serrate seta and six simple setae inferiorly, and one penicillate seta and four simple setae superiorly; merus 0.3 times as long as ischium, with one serrate seta and three simple setae inferiorly, and three simple setae superiorly; carpus similar to merus in length, with one simple seta and two serrate setae inferiorly; propodus oblong, 1.8 times longer than carpus, with two robust simple setae, one simple seta and several short simple setae on inferior margin, and one penicillate seta and one short simple seta at superodistal angle; dactylus rectangular, with four simple setae and one unguis distally. Pereopods 3–6 (Figs. 5B–5E) almost similar to pereopod 2; basis with tubercles superiorly except for pereopod 5.

Pleopods (Figs. 6A–6E) similar to each other; protopod ovoid to oblong, with one simple seta laterally, two coupling hooks medially; rami elongated ovoid, without plumose setae distally, except for pleopods 3 and 5; pleopod 2 with penicillate seta distally and one penicillate seta subdistally on exopod; pleopod 3 with three plumose setae on endopod and two plumose setae on exopod distally; pleopod 5 with one plumose seta on endopod and three plumose setae distally; appendix masculina not observed in pleopod 2.

Uropod (Fig. 6F), protopod rectangular, with one simple dorsal seta; rami with 7–10 simple setae along margin; endopod slightly longer than exopod, with 6–8 penicillate setae and 0–2 simple setae dorsally.

Remarks. In the 133 *gnathia* species, 13 species have paraocular ornamentations forming a ridge (Monod, 1926; Menzies, 1962; Schultz, 1966; Holdish & Harrison, 1980; Müller, 1993; Cohen & Poore, 1994; Pires, 1996; Tanaka, 2004; Kensley, Schotte & Poore, 2009; Ota, 2013; Song & Min, 2018; Shodipo et al., 2021). Among them, *G*. *obtusispina* **sp. nov.** most resembles two species, *G*. *lignophila* Müller, 1993 and *G*. *andrei* Pires, 1996, by having body integument covered by numerous tubercles (Müller, 1993; Pires, 1996). However, the new species can be easily distinguishable from these two species in that the frontal border of the cephalon is medially concave (*vs*. convex in the latter two species) and the pleotelson has rounded distal end (*vs*. acute distal end in the latter two species).

In the East Asia where the new species were collected, there are nine species characterized by the presence of tubercles on the cephalon and pereonites among 25 *Gnathia* species reported: *G*. *tuberculata* Richardson, 1909 from the Nanao, Japan; *G*. *derzhavini* Gurjanova, 1933 from the Askold Island, Russia; *G*. *schmidti* Gurjanova, 1933 from the Bay of Vladimir, Russia; *G*. *teruyukiae* Ota, 2011 from the Ishigaki Island, Japan; *G. rufescens* Ota, 2015 from the Okinawa Island Japan; *G*. *albipalpebrata* Ota, 2014 from the Okinawa-jima Island, Japan; *G*. *parvirostata* Ota, 2014 from the Ishigaki Island, Japan; *G*. *nubila* Ota & Hirose, 2009 from the Okinawa Island, Japan; and *G. dejimagi* Ota, 2014 from the Okinawa-jima Island, Japan (Boyko et al., 2008; Song & Min, 2018; Shodipo et al., 2021). Although *G*. *obtusispina* **sp. nov.** also represents this character state, this new species is easily distinguishable from the latter species by the combination of the following character states: (1) the body is covered with long setae; (2) the cephalon has a pair of remarkable tooth-like blunt paraocular ornamentations; (3) the frontal border of the cephalon is medially concave; (4) two inferior frontolateral processes are present ventrally; (5) the supraocular lobe is prominent and projecting upwards; (6) the dentate blade of the mandible is present and irregular; (7) pereonite 1 is not fused with cephalon dorsally and conspicuous; and (8) the apex of the pleotelson is rounded (Richardson, 1909; Gurjanova, 1933; Ota, 2011, 2015; Ota & Hirose, 2009).

Among the above-mentioned species, *G*. *obtusispina* **sp. nov.** is most similar to *G*. *tuberculata* by having inferior frontolateral processes and prominent supraocular lobes on cephalon, and mandible as long as half-length of the cephalon. However, the former differs from the latter in terms of the medially concave frontal border of the cephalon (*vs*. produced in the latter), presence of a tooth-like paraocular ornamentations (*vs*. absent in the latter), number of inferior frontolateral processes (two in the former *vs*. four in the latter), and rounded apex of the pleotelson (*vs*. acute in the latter) (Richardson, 1909).

Distribution. South Korea (the Yellow Sea)

Host. Unknown.

Etymology. The specific name, *obtusispina*, originates from the combination of Latin words *obtusus*, meaning “blunt” and *spina*, meaning “thorn”. This name refers to tooth-like paraocular ornamentation; gender feminine.

## Discussion

*Rocinela* is distributed worldwide. It particularly shows high-latitude diversity (Bruce, 2009). Indeed, based on marine ecoregions of the world by Spalding et al. (2007), 29 of 41 known *Rocinela* species have been reported from a temperate region (Table 1). Among the temperate species, 21 known species are recorded from the Pacific, with 12 species from the temperate Northern Pacific region, including seven species from the Far East. This means that the majority of *Rocinela* species have been described from the temperate Northern Pacific, so the region could be considered as diversity hotspot for the genus *Rocinela*. However, given that Bruce (2009) has mentioned that a significant number of undescribed species from the tropical western Pacific region is held at the Muséum national d’Histoire naturelle in Paris, the lack of attention on the *Rocinela* species was likely to negatively affect our knowledge of the *Rocinela* species diversity in trophic region. So, undescribed species can be discovered through further study in this region. While among 29 species are known from the temperate region, only two species, *R*. *angustata* and *R. belliceps*, show a broad distribution ranging from the Northwest to Northeast Pacific despite most *Rocinela* species having endemic distribution ranges (Richardson, 1904, 1905, 1909; Kussakin, 1979; Brusca & France, 1992). Considering that host-association times is correlated with the distribution range and that *Rocinela* species can attach to the host temporally, these endemic distribution ranges of *Rocinela* species might be due to their feeding strategy with temporary ectoparasites attaching to fishes in their particular life history (Bruce, 2009; Smit, Bruce & Hadfield, 2019). Although hosts of *R*. *angustata* and *R*. *belliceps* remain unknown, broad distribution ranges of these two species could be related to their host’s distribution patterns (Smit, Bruce & Hadfield, 2019).

**Table 1:** Summary of *Rocinela* species from the temperate region.

Species	Location	Biogeographic realms	References
*R*. *affinis* Richardson, 1904	Japan (Numazu)	TNP	Richardson (1904)
*R*. *americana* Schioedte & Meinert, 1879	USA (Maine)	TNA	Schioedte & Meinert (1879); Kussakin (1979)
*R*. *angustata* Richardson, 1904	USA (Bering Sea to Washington); Japan (Manazuru Zaki)	TNP	Richardson (1904); Brusca & France (1992)
*R*. *austeralis* Schioedte & Meinert, 1879	Chile (Straits of Magellan)	TSA	Schioedte & Meinert (1879)
*R*. *belliceps* (Stimpson, 1864)	USA (Alaska to California); Mexico (Clarion Island); Russia (Sea of Okhotsk)	TNP; TEP	Brusca & France (1992); Kussakin (1979)
*R*. *bonita* Bruce, 2009	New Zealand (Bounty Trough)	TA	Bruce (2009)
*R*. *cornuta* Richardson, 1898	USA (off Shumagin Bank)	TNP	Richardson (1898)
*R*. *danmoniensis* Leach, 1818	Europe (Bay of Biscay to Iceland)	TNA	Bruce (2009)
*R*. *dumerilii* (Lucas, 1849)	Mediterranean Sea	TNA	Bruce (2009)
*R*. *excavata* **sp. nov.**	South Korea (Chujado Island)	TNP	Present study
*R*. *garricki* Hurley, 1857	New Zealand (Cook strait)	TA	Hurley (1957)
*R*. *granulosa* Barnard, 1914	South Africa (Natal)	TSAf	Barnard (1914b)
*R*. *Japonica* Richardson, 1898	Japan (Hakodate Bay)	TNP	Richardson (1898)
*R*. *juvenalis* Menzies & George, 1972	Peru (off Peru)	TSAm	Bruce (2009)
*R*. *kapala* Bruce, 1988	Australia (New South Wales)	TA	Bruce (1988)
*R*. *laticauda* Hansen, 1897	Mexico (off Acapulco); USA (California)	TEP; TNP	Brusca & France (1992)
*R. leptopus* Bruce, 2009	New Zealand (Pagasus Bay)	TA	Bruce (2009)
*R*. *lukini* Vasina, 1993	Sea of Okhotsk	TNP	Vasina (1993)
*R*. *maculata* Schioedte & Meinert, 1879	Russia (Vladivostok)	TNP	Schioedte & Meinert (1879)
*R*. *niponia* Richardson, 1909	Japan (Sado Island); South Korea (Chujado Island)	TNP	Richardson (1909); Kim & Yoon (2020)
*R*. *ophthalmica* Milne Edwards, 1840	Italy (Sicily)	TNA	Bruce (2009)
*R*. *pakari* Bruce, 2009	New Zealand (Chatham Rise)	TA	Bruce (2009)
*R*. *patriciae* Brasil Lima, 1986	Brazil (off Rio Grande do Sul)	TSAm	Bruce (2009)
*R*. *propodialis* Richardson, 1905	USA (Washington)	TNP	Richardson (1905)
*R*. *resima* Bruce, 2009	New Zealand (Christabel Sea Mount)	TA	Bruce (2009)
*R*. *satagia* Bruce, 2009	New Zealand (Chatham Rise)	TA	Bruce (2009)
*R*. *sila* Hale, 1925	Australia (Adelaide)	TA	Hale (1925)
*R*. *tridens* Hatch, 1947	USA (Washington)	TNP	Hatch (1947)
*R*. *tropica* Brasil Lima, 1986	Brazil (Espírito Santo)	TSAm	Bruce (2009)
*R*. *tuberculosa* Richardson, 1898	Mexico (Baja California)	TNP	Richardson (1898)

**Note:**

TA, Temperate Australasia; TEP, Temperate Eastern Pacific; TNA, Temperate Northern Atlantic; TNP, Temperate Northern Pacific; TSAf, Temperate Southern Africa; TSAm, Temperate Southern America.

Fifty-six and 76 species of 133 known *Gnathia* species have been reported from a temperate region and tropical region, respectively (Table 2; Song & Min, 2018; Shodipo et al., 2021). Only two species, *G*. *fragilis* Schultz, 1977 and *G. tuberculosa* (Beddard, 1886), are from the Southern Ocean, Antarctic (Monod, 1926; Schultz, 1977). According to the marine ecoregions of the world, the Central Indo-Pacific (with 47 species) is thought to be the most diverse hotspot of *Gnathia* (Shodipo et al., 2021). After the Central Indo-Pacific, the second-most rich species of 18 species have been reported from the temperate Northern Pacific that includes the study area of the present study. Consequently, the temperate Northern Pacific is considered to be the second most diverse hotspot following the Central Indo-Pacific. Within the temperate Northern Pacific, the Far East, from which 11 *Gnathia* species are recorded, could be regarded as a representative hotspot. While looking for substrate types from which *Gnathia* species are collected, most temperate species have been collected from soft substrates such as mud, silt, and sandy flats in contrast to tropical *Gnathia* species reported from coral-reef habitats (Cohen & Poore, 1994; Svavarsson & Bruce, 2012). This result is a mismatch to the general knowledge that gnathiid species prefer coral reef-associated habits (Cohen & Poore, 1994; Santos & Sikkel, 2019; Smit, Bruce & Hadfield, 2019; Svavarsson & Bruce, 2012, 2019). Furthermore, the feature of the substratum strongly affects the distribution of gnathiids, and each species has a different habitat depending on its life stages (Smit, Bruce & Hadfield, 2019). Taken all together, the life history of *Gnathia* species is likely to differ depending on whether they live in a temperate or a tropic region (Santos & Sikkel, 2019). However, further study about the substratum preference between temperate and tropic *Gnathia* species is needed because most ecological studies of these species have been conducted from coral reef-associated habitats (Grutter, Morgan & Adlard, 2000, Grutter et al., 2018; Santos & Sikkel, 2019; Smit, Bruce & Hadfield, 2019; Shodipo et al., 2021). Additionally, although most *Gnathia* species are known as endemic, two species, *Gnathia calmani* Monod, 1926 and *Gnathia nasuta* Nunomura, 1992, have wide distributions ranging from the tropic to the temperate region (Monod, 1926; Holdish & Harrison, 1980; Nunomura, 1992; Ota, 2013). Another two species, *G. grandilaris* Coetzee et al., 2008 and *G. trimaculata* Coetzee et al., 2009, have been reported only from the Central Indo-Pacific, and also show a wide geographical distribution ranging from Australia to Japan (Coetzee et al., 2008, 2009; Ota & Hirose, 2009). According to Shodipo et al. (2021), the long-distance dispersal of some *Gnathia* species was facilitated by their host that had a wide movement radius in a short period of time (*e.g.*, sharks). Considering wide movement radii of hosts such as sharks and rays in *G*. *grandilaris* and *G*. *trimaculata*, the two species showing wide distribution ranges, *G*. *calmani* and *G*. *nasuta*, also could be parasites of hosts having wide movement radii (Coetzee et al., 2008, 2009; Shodipo et al., 2021).

**Table 2:** Summary of *Gnathia* species from the temperate region.

Species	Location	Biogeographic realms	References
*G*. *africana* Barnard, 1914	South Africa (Cape Town)	TSAf	Barnard (1914a); Monod (1926); Smit, As & Basson (1999); Smit, Van As & Basson (2002)
*G*. *albescens* Hansen, 1916	Denmark (Foroe Island)	TNA	Hansen (1916)
*G*. *andrei* Pires, 1996	Brazil (Ubatuba continental slope)	TSAm	Pires (1996)
*G*. *brachyuropus* Monod, 1926	New Zealand (Akaroa, Lyttelton)	TA	Monod (1926)
*G*. *brucei* George, 2003	USA (North Carolina)	TNA	George (2003)
*G*. *bungoensis* Nunomura, 1982	Japan (Saeki Bay)	TNP	Nunomura (1982)
*G*. *calamitosa* Monod, 1926	Australia (New South Wales)	TA	Monod (1926)
*G*. *calmani* Monod, 1926	Australia (Heron Island; Victoria)	CIP; TA	Monod (1926)
*G*. *campontus* Cohen & Poore, 1994	Australia (Bass Strait)	TA	Cohen & Poore (1994)
*G*. *capillata* Nunomura & Honma, 2004	Japan (Sado Island)	TNP	Nunomura & Honma (2004)
*G*. *clementensis* Schultz, 1966	USA (California)	TNP	Schultz (1966)
*G*. coronadoensis Schultz, 1966	USA (Coronado canyon)	TNP	Schultz (1966)
*G*. *dentata* (G. O. Sars, 1872)	Norway (Hardangerfijord)	TNA	Monod (1926)
*G*. *derzhavini* Gurjanova, 1933	Russia (Askold Island)	TNP	Gurjanova (1933)
*G*. *disjuncta* Barnard, 1920	South Africa (Cape Town)	TSAf	Monod (1926)
*G*. *epopstruma* Cohen & Poore, 1994	Australia (Bass Strait)	TA	Cohen & Poore (1994)
*G*. *fallax* Monod, 1926	Spain (Bay of Biscay)	TNA	Monod (1926)
*G*. *gurjanovae* Golovan, 2006	Russia (Peter the Great Bay)	TNP	Golovan’ (2006)
*G*. *hirsuta* Schultz, 1966	USA (California)	TNP	Schultz (1966)
*G*. *illepidus* (Wagner, 1869)	Mediterranean Sea (Italy, Monaco)	TNA	Monod (1926)
*G*. *incana* Menzies & George, 1972	Peru (off Peru)	TSAm	Menzies & George (1972); Cohen & Poore (1994)
*G*. *inopinata* Monod, 1925	Mediterranean Sea (Italy, Monaco)	TNA	Monod (1926)
*G*. *iridomyrmex* Cohen & Poore, 1994	Australia (Victoria)	TA	Cohen & Poore (1994)
*G*. *koreana* Song & Min, 2018	South Korea (Geomundo Island)	TNP	Song & Min (2018)
*G*. *lacunacapitalis* Menzies & George, 1972	Peru (off Peru)	TSAm	Menzies & George (1972); Cohen & Poore (1994)
*G*. *maxillaris* (Montagu, 1804)	England (Cornwall)	TNA	Monod (1926)
*G*. *mulieraria* Hale, 1924	Australia (Gulf St. Vincent)	TA	Hale (1924)
*G*. *mutsuensis* Nunomura, 2004	Japan (Asamushi)	TNP	Nunomura (2004)
*G*. *mystrium* Cohen & Poore, 1994	Australia (Bass Strait)	TA	Cohen & Poore (1994)
*G*. *nasuta* Nunomura, 1992	Japan (off Tomioka; Amai; Keramal Okinawa islands)	CIP; TNP	Nunomura (1992)
*G*. *nkulu* Smit & Van As, 2000	South Africa (Port Alfred)	TSAf	Smit & Van As (2000)
*G*. *notostigma* Cohen & Poore, 1994	Australia (Bass Strait)	TA	Cohen & Poore (1994)
*G*. *obtusispina* **sp. nov.**	South Korea (Hongdo Island)	TNP	Present study
*G*. *odontomachus* Cohen & Poore, 1994	Australia (Victoria)	TA	Cohen & Poore (1994)
*G*. *oxyuraea* (Lilljeborg, 1855)	North Sea	TNA	Monod (1926)
*G*. *panousei* Daguerre de Hureaux, 1971	Morocco	TNA	Boyko et al. (2008)
*G*. *pantherina* Smit & Basson, 2002	South Africa (Jeffreys Bay)	TSAf	Smit & Basson (2002)
*G*. *phallonajopsis* Monod, 1925	Mediterranean Sea (France, Italy, Monaco, Sapin)	TNA	Monod (1926)
*G*. *pilosus* Hadfield, Smit & Avenant-Oldewage, 2008	South Africa (Sheffield Beach, Tinley Manor)	TSAf	Hadfield, Smit & Avenant-Oldewage (2008)
*G*. *productatriedns* Menzies & Barnard, 1959	USA (California)	TNP	Menzies & Barnard (1959)
*G*. *prolasius* Cohen & Poore, 1994	Australia (Bass Strait)	TA	Cohen & Poore (1994)
*G*. *rectifrons* Gurjanova, 1933	Russia (East Sea)	TNP	Gurjanova (1933)
*G*. *ricardoi* Pires, 1996	Brazil (Ubatuba continental slope)	TSAm	Pires (1996)
*G*. *sanrikuensis* Nunomura, 1998	Japan (Otsuchi Bay)	TNP	Nunomura (1998)
*G*. *schmidti* Gurjanova, 1933	Russia (Bay of Vladimir)	TNP	Gurjanova (1933)
*G*. *serrulatifrons* Monod, 1926	Mediterranean Sea	TNA	Monod (1926)
*G*. *sifae* Svavarsson, 2006	New Zealand (Bay of Plenty)	TA	Svavarsson (2006)
*G*. *spongicola* Barnard, 1920	South Africa (False Bay)	TSAf	Monod (1926)
*G*. *steveni* Menzies, 1962	USA (California)	TNP	Menzies (1962)
*G*. *stigmacros* Cohen & Poore, 1994	Australia (Bass Strait)	TA	Cohen & Poore (1994)
*G*. *teissieri* Cals, 1972	Spain (Bay of Biscay)	TNA	
*G*. *tridens* Menzies & Barnard, 1959	USA (California)	TNP	Menzies & Barnard (1959)
*G*. *trilobata* Schultz, 1966	USA (Coronado)	TNP	Schultz (1966)
*G*. *tuberculata* Richardson, 1909	Japan (Nanoa)	TNP	Richardson (1909)
*G*. *ubatuba* Pire, 1996	Brazil (Ubatuba continental slope)	TSAm	Pires (1996)
*G*. *venusta* Monod, 1925	Mediterranean Sea (Monaco)	TNA	Monod (1926)
*G*. *vorax* (Lucas, 1849)	Mediterranean Sea (Algeria, Bay of Biscay, Cape Bojador)	TNA	Monod (1926)

**Note:**

CIP, Central Indo-Pacific; TA, Temperate Australasia; TEP, Temperate Eastern Pacific; TNA, Temperate Northern Atlantic; TNP, Temperate Northern Pacific; TSAf, Temperate Southern Africa; TSAm, Temperate Southern America.

## Conclusion

The present study of Korean ectoparasitic isopods revealed high species diversity of *Rocinela* and *Gnathia* species in the temperate Northern Pacific region by the discovery of two new species, *Rocinela excavata* **sp. nov.** and *Gnathia obtusispina* **sp. nov.** The two new species are the species records for the 13^th^ *Rocinela* species and the 19^th^ *Gnathia* species in this region, respectively. Our investigation on the geographical distributions of known *Rocinela* and *Gnathia* species indicated that the temperate Northern Pacific has the most *Rocinela* species and the second most *Gnathia* species in the regional species richness of each genus. It also showed that even if both genera indicate great diversity in the western Pacific, *Rocinela* species reveal high-latitude diversity while *Gnathia* species represent low-latitude diversity, particularly in the Central Indo-Pacific region.

## Additional Information and Declarations

### Field Study Permissions

The following information was supplied relating to field study approvals (*i.e*., approving body and any reference numbers): Field experiments were approved by Ministry of Environment (MOE) of the Republic of Korea (project number: NIBR201902204; NIBR202102204).

### Data Availability

The following information was supplied regarding data availability: The raw data are line drawings of two new species and the morphological character.

### New Species Registration

The following information was supplied regarding the registration of a newly described species: *Rocinela excavata* species LSID: urn:lsid:zoobank.org:act:9A4CC86D-6930-4FC6-9FC9-DBF105A2B285 *Gnathia obtusispina* species LSID: urn:lsid:zoobank.org:act:3219C531-9A69-4805-B16D-5B14AF1B9B61 Publication LSID: urn:lsid:zoobank.org:pub:7A53937A-F2EB-49C7-B8DA-F0AA36241310.
